# Conjugated Linoleic Acid and Cholesterol Oxidative Products Generated in Hot Boned Beef *Semimembranosus* Muscle as Affected by Rigor Temperature, Ageing and Display Time

**DOI:** 10.3390/foods9010043

**Published:** 2020-01-03

**Authors:** Tanyaradzwa E. Mungure, E. John Birch, Eric N. Ponnampalam, Ian Stewart, Isam A. Mohamed Ahmed, Fahad Y. Al-Juhaimi, Alaa El-Din A. Bekhit

**Affiliations:** 1 Department of Food Science, University of Otago, P.O. Box 56, Dunedin 9054, New Zealand; john.birch@otago.ac.nz; 2 Animal Production Sciences, Agriculture Victoria Research, Department of Jobs, Precincts and Regions, AgriBio, Bundoora, Victoria 3083, Australia; Eric.Ponnampalam@agriculture.vic.gov.au; 3 Chemistry Department, University of Otago, P.O. Box 56, Dunedin 9054, New Zealand; ian.stewart@otago.ac.nz; 4 Department of Food Science and Nutrition, College of Food and Agricultural Sciences, King Saud University, Riyadh 11451, Saudi Arabia; iali@KSU.EDU.SA (I.A.M.A.); faljuhaimi@ksu.edu.sa (F.Y.A.-J.)

**Keywords:** ageing, beef semimembranosus muscle, Cholesterol CLA, oxidation, rigor temperature

## Abstract

The present study investigated the effect of processing parameters comprising rigor temperature, ageing and display time on conjugated linoleic acid (CLA) concentrations, stability and the development of cholesterol oxidation products in hot boned beef *semimembranosus* muscles. Meat samples, having attained rigor mortis at 5 °C and 25 °C, were vacuum packed and aged for 7 and 14 days and then displayed under aerobic conditions for 7 days at 4 °C. Lipid was extracted at each time interval then ^1^H NMR and GC-FID were used for CLA quantification. The cholesterol oxidation products (COPs) were separated from lipids via column chromatography and derivatized for GC-FID. The CLA content was not affected by the rigor temperature, ageing and display time (*p* > 0.05). The cholesterol oxidative stability was not affected by rigor temperature (*p* > 0.05) but was affected by ageing and display time (*p* < 0.05). The COPs, 7α- and 7β-hydroxycholesterol, and 7-ketocholesterol were positively identified and their quantities increased with ageing and display time (*p* < 0.05). These results demonstrate that the production of COPs in semimembranosus muscle was significantly altered by the ageing and display time parameters but not by the rigor temperature used in this study.

## 1. Introduction

Meat and processed meat products have been reported to contribute up to 25%–30% of total dietary conjugated linoleic acid (CLA) intake in the Western world [1]. CLA is a collective term for a group of positional and geometric isomers of linoleic acid (c9, c12-octadecadienoic acid). They have two double bonds separated by a single carbon bond, hence conjugated bonds [2]. Initially, CLAs were only of interest to rumen microbiologists who studied the most abundant isomer cis-9, trans-11 CLA as an intermediate in the biohydrogenation of linoleic acid, until Pariza and Hargraves [3] discovered antimutagenic substances in pan-fried hamburgers that were effective inhibitors of benzopyrene-initiated mouse epidermal neoplasia. The antimutagenic substances were identified as CLAs. Through the years, the list of known positive physiological and nutritional benefits from dietary CLA consumption has increased. These positive benefits include anticarcinogenic and antithrombotic effects, cardiovascular disease interactions, and their potential support of the immune system and bone health, to name a few [2,4]. Previous work on CLAs in red meat have focused predominantly on the effect of dietary regimes, supplementation, age and sex, with limited work performed on the implications of processing parameters on CLA concentrations and cholesterol oxidation (i.e., interactions of rigor temperature, ageing and displaying times) [5,6,7,8].

Cholesterol (cholest-5-en-3β-ol), an important component in several metabolic processes, is a polycyclic molecule with a double bond between carbons 5 and 6 [9]. The important biological processes involving cholesterol include the synthesis of steroids and steroidal hormones, and the biosynthesis of bile and bile acid salts. Cholesterol also plays a significant role in cellular membrane architecture, affecting its stability, permeability, and fluidity [10,11]. The biosynthesis of cholesterol occurs in animal tissues. In mammals, biosynthesis is performed mainly in the liver, adrenal gland, ovaries and testis [12]. Some of the cholesterol found in food products of animal origin is absorbed from dietary intake [9]. Cholesterol is excreted mainly from the bile and is unesterified on release [12]. The cholesterol absorbed from dietary intake is formed as a lipoprotein complex (e.g., chylomicron) and travels in the blood, which can be removed from the circulatory system by the liver as a component of chylomicron remnants [10].

Cholesterol, due to its unsaturated double bond, is susceptible to oxidation, leading to the formation of oxysterols/cholesterol oxidation products (COPs) in the presence of light, oxygen, heat, free radicals and several other factors [9,13,14]. The COPs are sterols like cholesterol, but contain an extra oxygen functional group, which can be a ketone (C=O), hydroxyl (-OH), epoxide (C-O-C, glycidyl ether ring) or hydroperoxide (-OOH) [15] on the nucleus of the sterol and in some cases a hydroxyl on the side chain of the molecule.

Although steroidal cholesterol is an essential metabolite required for important biological functions in live animals, the oxidation products of cholesterol in meat have been reported to be involved in several chronic and degenerative diseases [16,17]. They have been reported to have potential cytotoxic, atherogenic, carcinogenic and mutagenic effects on human health [18]. The level of cholesterol oxidation can be linked to the processing and manufacturing conditions, such as temperature and storage length, as well as antioxidant availability [19]. The COPs have been reported to inhibit cholesterol biosynthesis, leading to its limited bioavailability [18]. The most predominant oxidation derivatives reported from cholesterol in food products include 7-ketocholesterol (7-KC), 7α-hydroxycholesterol, 7β-hydroxycholesterol (7α- and 7β-HC), 20-hydroxycholesterol, 25-hydroxycholesterol, cholesterol-5α, 6α-epoxide, cholesterol-5β, 6β-epoxide and cholestane-3β,5α,6β-triol [20]. Due to the presence of the unsaturated double bond on carbon 5 of cholesterol, a wide range of COPs can be produced endogenously or exogenously through various reaction pathways [20]. The COPs can be produced by chemical autoxidation and photosensitized and enzymatic oxidation. The most frequently observed mechanism in food products is autoxidation. Photosensitized oxidation is not as common as autoxidation, and enzymatic oxidation is the least likely to occur [21].

To the authors’ knowledge, the effects of rigor temperature and ageing on the oxidative stability of CLA and cholesterol in hot boned beef have not been reported. Therefore, the objective of this study was to assess the effects of rigor temperature, ageing of meat under vacuum and display time on CLA concentration, cholesterol concentration, and oxidative stability of cholesterol in hot boned beef semimembranosus (SM) muscle. Previous studies have shown a decline in polyunsaturated fatty acids (PUFAs) due to oxidative processes [22], but not much work has been done to establish the particular effects of display time on the development of COPS (qualitatively and quantitatively) and the oxidation of CLA. This study also explored whether the effect of hot boned beef entering rigor mortis at a higher temperature would trigger an increase in oxidative processes, thereby affecting cholesterol and CLA. The study also applied multi-faceted CLA analytical approaches, using traditional and novel NMR spectroscopy methods for data analysis and validation.

## 2. Materials and Methods

### 2.1. Reagents and Chemicals

The chemicals and reagents were all of analytical grade (Sigma grade ≥95% purity). The cholesterol and cholesterol oxide standards of cholesterol (5-cholesten-3β-ol), 5α-cholestane, 6-ketocholesterol (5α-cholestan-3β-ol-6-one), α-epoxide (5α, 6α-epoxycholestan-3β-ol), β-epoxide (5β, 6β-epoxycholestan-3β-ol) and 20α-hydroxycholesterol were from Sigma Aldrich Inc. (St Louis, MO, USA). Potassium hydroxide was purchased from Thermo Fisher Scientific (Waltham, MA, USA). Butylated hydroxytoluene (BHT), Celite 545, dicalcium phosphate dehydrate (CAHPO_4_ × 2H_2_O), pyridine, BSTFA (N,O-bis(trismethylsilyl) trifluoroacetamide) and TMCS (trimethylchlorosilane) (BSTFA + 1% TMCS), and 1,4-dioxane were also purchased from Sigma Aldrich Inc. (St Louis, MO, USA). Methanol and diethyl ether were from Riedel-de Haen, Seelze, Germany. Trichloromethane, sulphuric acid, fydrochloric acid (32%), hexane, ethyl acetate, and acetone were from Fisher Scientific (Poole, England). Sodium chloride was from BDH Chemicals (Poole, England).

### 2.2. Meat Sampling and Processing

Six beef SM muscles from 6 Hereford heifers (18 months old) raised on pasture, with a carcass weight of 278.9 ± 28.4 kg, were purchased from an export licensed meat processing plant (Alliance Group Limited, Pukeuri Station, Oamaru, New Zealand). After exsanguination, the carcasses were electrically stimulated [square mono wave 80 V, 25 s], hot-boned 45 min after slaughter, and processed within 2 h postmortem. The left and right topsides from each animal were separated and each muscle was further cut into 4 blocks. The blocks were randomly distributed to 2 different rigor temperature treatment incubators, 5 and 25 °C. The pH decline was monitored every hour until the ultimate pH was attained to determine rigor mortis (data not shown). The ultimate pH was achieved after 9 and 24 h post-mortem for samples incubated at 25 and 5 °C, respectively. Once the samples reached ultimate pH, they were vacuum packed, transferred into a chiller set at 2 °C and aged for a further 7 or 14 days. After each designated ageing time, the meat blocks were sliced into about 2.5 cm samples that were placed in polystyrene trays and wrapped with oxygen permeable polyvinylchloride film (O_2_ permeability >2000 mL m^−2^ atm^−1^. 24 h^−1^ at 25 °C) (AEP FilmPac Ltd., Auckland, New Zealand). The samples were displayed under cool fluorescent light (1076 lux) for 7 days of display time under aerobic conditions at 4 °C. The slices were sampled at the start of the display time (initial) and after 7 days of display (end) for the determination of cholesterol, COPs and CLA concentrations, as elaborated below.

### 2.3. Analysis of Cholesterol and Its Oxidative Stability

Lipids were extracted from the samples using a modified Folch et al. [23] extraction method. About 10 g of meat, 50 µL of BHT (7.2%), and 50 mL solution of trichloromethane–methanol (2:1 *v*/*v*, Folch solution 1) was added to a 100 mL capped test tube. The samples were dispersed using a Polytron at 14,000 rpm for 30 s. The meat homogenate was left overnight and filtered through Whatman filter paper number 1 into a 100 mL cylinder with a glass stopper. The residue was washed with 10 mL of Folch solution 1. A 15 mL of 0.90% NaCl solution was added and mixed by vigorous shaking. The contents were transferred into a separation funnel, and the cylinder was rinsed with 2 mL trichloromethane, methanol and water mixture (3:48:49, Folch solution 2). The washings were then added to the separation funnel. The two phases were left to separate, and the bottom layer (trichloromethane layer) was carefully siphoned into an evaporation flask. Solvent removal (trichloromethane) was performed with a rotary evaporator (VacuuBrand GMBH + Co KG, Wertheim Germany) in a warm water bath at 30 °C. The dried lipid collected from the rotary evaporator process was dissolved in hexane to a concentration of 0.1 g lipid per mL of hexane for column chromatography.

Column chromatography was set up as described by Li et al. [24] for the preparation of cholesterol and its oxidation products. A mixture of silicic acid (100 mesh), Celite 545, and CaHPO_4_. 2H_2_O (10:9:1 *v*/*v*/*v*) in trichloromethane was prepared and packed into a glass column with dimensions of 22 mm by 300 mm with glass wool on the base. The column was washed with 20 mL of solvent 1 (hexane: ethyl acetate, 9:1 *v*/*v*). The beef lipid sample from above (1 mL) was loaded onto the column. A 4 mL aliquot of solvent 2 (hexane: ethyl acetate, 4:1 *v*/*v*) was run through the column to wash out the neutral lipids. After completion, 40 mL of solvent 3 (acetone: ethyl acetate: methanol 10:10:1 *v*/*v*/*v*) was passed through the column at 1 mL/min to elute cholesterol and its oxidation products (COPs) which were collected in 50 mL glass vials. The solvent was evaporated using a steady flow of nitrogen. Before loading the next sample, the column was washed with 20 mL of methanol to elute the remaining phospholipids. The collected cholesterol and its oxidation products were derivatized with 300 µL of pyridine and 100 µL of (*N.O*-Bis(trimethylsilyl)trifluoroacetamide +1% Trimethylchlorosilane) BSTFA+ 1% TMCS. The samples were left to react overnight before conducting gas chromatography—flame ionization detection (GC-FID) analysis further described below.

### 2.4. GC-FID Analysis

Cholesterol and its oxidation products were separated using an HP-5 column of 30 m × 320 µm × 0.25 µm containing 5% phenyl methyl silicon (Hewlett Packard, Avondale, PA, USA). The gas chromatographic system consisted of a Hewlett Packard 6890N GC equipped with an autosampler (HP7673) and ChemStation integration system (all Hewlett Packard, Avondale, PA, USA). The column oven was held at an initial temperature of 180 °C and increased at a rate of 8 °C min^−1^ to 260 °C. It was then further increased to 280 °C at 2 °C min^−1^ and held for 15 min. Both the injector and flame ionization detector ports were at 290 °C. The carrier gas flow (hydrogen) was maintained at a constant 1.2 mL min^-1^ flow throughout the temperature program.

Cholesterol and its oxidation product peaks were identified by retention time matching with the authentic derivatized standards (NuCheck Prep, Elysian, Minnesota and Sigma, St. Louis, MO, USA). The quantitation of cholesterol and its oxidation products were calculated using an internal standard, 5α-cholestane, added during sample preparation.

### 2.5. Fatty Acid Methyl Esterification (FAME) of Lipid from Beef SM Muscle

A modified method of that which Van Wijngaarden [25] used for fatty acid methylation has been reported in detail in Mungure et al. [22]. The extracted lipid (15 mg), using a modified Folch et al. [23] extraction method, was weighed into a test tube with a Teflon liner cap, and 10 mL of hexane was added and thoroughly dissolved. Of this mixture, 2 mL was pipetted into a new test tube. An aliquot of 2 mL, 0.5 N KOH in methanol was added and vortexed (Chiltern MT, Chiltern Scientific Instrumentation Ltd., England) for 1 min. The mixture was left to saponify at 80 °C using a heating block for 20 min. After cooling the mixture at room temperature, 2 mL of diethyl ether and 5 mL of milli-Q water was added. The solution was gently shaken and left to settle into 2 separate phases. The upper layer constituting diethyl ether was discarded with aqueous bottom layer retained. The aqueous layer was acidified by drops of HCL (32%) until the litmus paper turned red. Diethyl ether (2 mL) and 1 mL BF_3_ (in 14% methanol) were subsequently added. The mixture was heated at 80 °C for 20 min. The solution was left at room temperature to cool off and 5 mL of saturated NaCl was added and vortexed for 1 min. After the two phases settled, the upper layer was pipetted into a 1.5 mL vial for GC-FID analysis.

The analysis of the FAMEs was performed on an Agilent PH6890 using a BPX-70 capillary column (100 × 0.22 i.d; 0.25 µm films, SGE, Melbourne, Australia). The GC was equipped with an HP7673 autosampler and ChemStation integration (all Hewlett Packard, Avondale, PA, USA). The injector and detector port temperatures were 250 °C. The initial temperature of the column oven was set at 165 °C and held for 52 min and increased to a final temperature of 210 °C at 5 °C min^−1^. The final temperature of 210 °C was held for 59 min. The other parameters of the run were as follows: carrier flow gas, helium 1.0 mL min^−1^, linear gas velocity 0.2 m s^−1^, and inlet split ratio 30:1. A composite standard of FAMEs was made from commercially available methyl esters (NuCheck Prep, Elysian, Minnesota and Sigma, St. Louis, MO, USA) and the data were analyzed with HP ChemStation computer software (Hewlett Packard, Agilent Technologies Inc., Avondale, PA, USA).

### 2.6. ^1^H NMR CLA Identification and Quantitative Measurements

The CLA concentrations in beef lipid were analyzed using the methods of Prema et al. [26] and Manzano et al. [4] with modifications on the selected internal standard used. The ^1^H NMR spectra were acquired using a Varian/Agilent 400 MHz NMR spectrometer with a Oneprobe and Varian /Agilent 7600AS robot (Palo Alto, CA, USA) with a magnet of 9.4T; CDCl_3_ was used as the solvent and the residual protons as an internal reference (7.26 ppm). The spectra were obtained at 25 °C with a spectral window of 9.50 to 0.00 ppm, 80° pulse width, 65,536 data points, 128 scans at a spectral width of 6400 Hz, a relaxation delay of 1.4 s and an acquisition time of 2 s. An exponential line broadening, automatic phase and base correction were applied to each spectrum.

**Figure 1:**
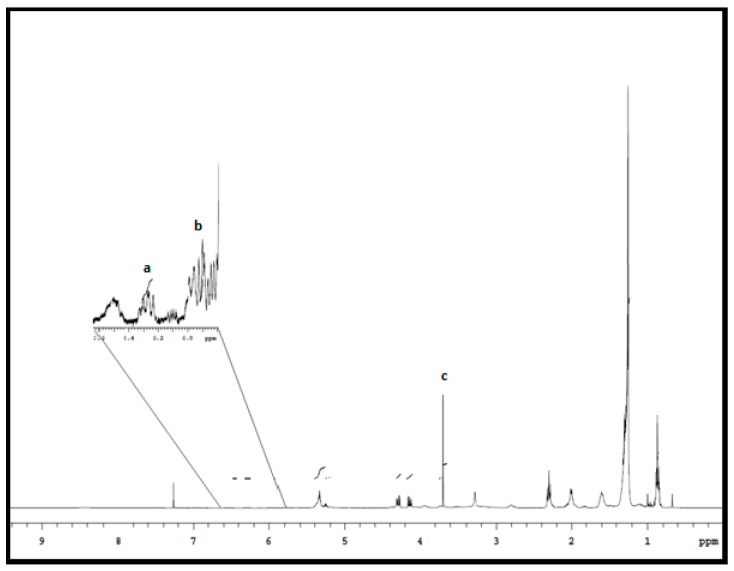
^1^H NMR spectrum for hot boned beef semimembranosus (SM) muscle lipid extract. The region 6.60–5.98 ppm was enlarged to visualize the integrated chemical shifts in analyzing the conjugated linoleic acid (CLA) concentration in the sample. CLA concentrations were obtained by averaging the integrated chemical shifts (peak a and b) shown above. Chemical shift (peak c) is the internal standard 1,4-dioxane used for quantitation of CLA in the study.

For quantitative analysis, a known quantity of 1,4-dioxane (C_4_H_8_O_2_) (0.0806 mg) was added as an internal standard to 650 µL of CDCl_3_ in each NMR tube along with the beef lipid from a stock solution of 6.2 mg in 50 mL CDCL_3_. The conjugated linoleic acid concentrations in the samples were quantitated by averaging the integrated areas at 6.28 and 5.92 ppm as normalized to the single methylene signal of the internal standard at 3.70 ppm. The CLA content (mg g^−1^ beef lipid) in each sample was calculated using the equation below:

$$\begin{array}{cc} \mathrm{Total}\;\mathrm{CLA}\;\mathrm{content}\;(\mathrm{mg}/g\;\mathrm{beef}\;\mathrm{lipid}) & =(\frac{\mathrm{Icla}}{\mathrm{IDioxane}})\times (\frac{\mathrm{Hcla}}{\mathrm{HDioxane}})\\ & \times (\frac{\mathrm{MWTAG}}{\mathrm{MWDioxane}})\times (\frac{M\mathrm{is}}{M\mathrm{Bl}}) \end{array}$$

where I_cla_ is the integrated average area for chemical shifts for CLA peaks a and b (see Figure 1), I_Dioxane_ is the signal area for internal standard 1,4-dioxane, H_cla_ is the number of protons of the CLA signal (1H), H_Dioxane_ is the number of protons for 1,4-dioxane methylene, MW_TAG_ is the molecular weight of fatty acid (FA) triacylglycerol (864.5 g mol^−1^), MW_Dioxane_ is the molecular weight of 1,4-dioxane (88.11 g mol^−1^), *M*_is_ is the mass of internal standard in the NMR tube (0.0806 mg) and *M*_Bl_ is the mass of beef lipid in the NMR tube (g).

It is important to consider the high possibility of 3 fatty acid (FA) groups esterifying on the same glycerol moiety [26], so the molecular weight of triacylglycerol reflects the maximum number of possible FAs that can attach to the glycerol [27]. The calculated MW_TAG_ of 864.5 g mol^−1^ was determined to represent an average of fatty acid composition in beef [27,28].

### 2.7. Statistical Analysis

The data for CLA analysis and cholesterol and oxidation products were tabulated using Microsoft Excel spreadsheets. The analysis of variance (ANOVA) was carried out using the Minitab (version 16.2.4) GLM repeated measurement procedure to examine the effects of rigor temperature (5 and 25 °C), ageing time (7 and 14 days) on the measured parameters at the start and end of display time (7 days). The samples were measured in duplicate, and significant differences among the mean values were determined at 5% significance level and the means were separated using Tukey’s test. 

## 3. Results and Discussion

### 3.1. Effect of Rigor Temperature, Ageing and Display Time on CLA Concentration in Hot Boned Beef SM Muscle

Figure 1 displays the ^1^H NMR spectrum of a beef SM muscle lipid sample with the internal standard, 1,4-dioxane. The important signals emanating from the protons of the conjugated double bonds of CLA appear between 6.5 ppm to 5.0 ppm [4,25]. The integral peaks on a ^1^H NMR spectrum are directly proportional to the number of resonant nuclei, and signals with chemical shifts between 6.28 and 5.93 ppm (Peaks a and b, respectively) are unique to CLAs.

**Figure 2:**
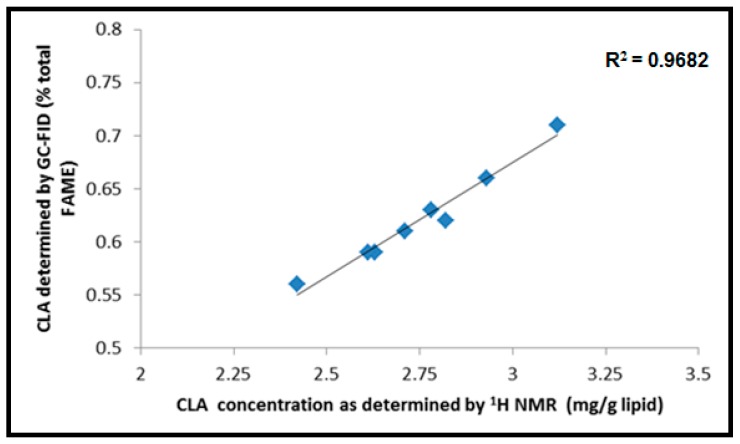
Correlation between total concentrations of CLA as determined by ^1^H NMR and GC-FID.

These resonances do not overlap with the chemical shifts representing other fatty acids, simplifying the quantitation of total CLA content in meat lipid using ^1^H NMR [25,29]. The chemical shift at 3.70 ppm (Peak c) was for the methylene proton signal of internal standard 1,4-dioxane. It is important to note that the most common CLA isomers—trans-10, cis-12 CLA and cis-9, trans-11 CLA—in beef SM meat have the same chemical shift observed through ^1^H NMR analysis. The obtained results, therefore, refer to the total CLA content in the beef SM muscle samples. This also applied to GC-FID, as all the CLA isomers co-eluted, thereby having the same retention times. The analysis of CLA concentration from both analytical techniques showed a high correlation (R^2^ = 0.9682, Figure 2) and was in agreement with the results reported by Manzano et al. [4] on beef lipids and the results of Prema et al. [26] on selected Canadian Cheeses.

**Figure 3:**
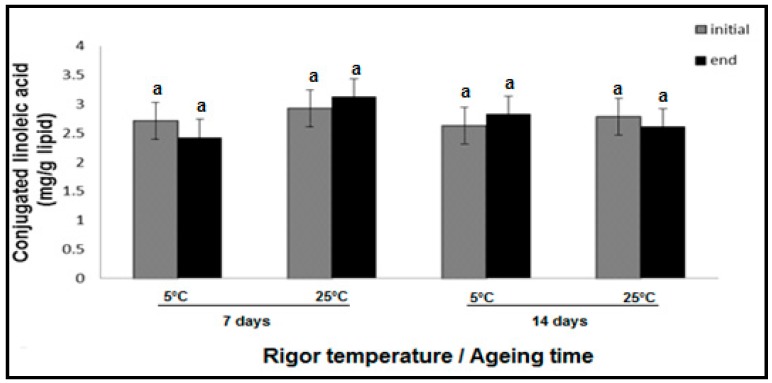
CLA concentrations in hot boned beef SM muscle lipid determined by ^1^H NMR. Bars represent Mean ± SEM (*n* = 6). Bars with different superscripts are significantly different at (*p* > 0.05). Key: “initial” CLA concentrations were obtained at the start of display time. The “end” CLA concentrations were obtained at the completion of 7 days of display time.

The rigor temperature did not have an effect on CLA oxidation (*p* > 0.05, Figure 3). The ageing time under vacuum packaging from 7 to 14 days and 7 days of display time also had no effect on CLA stability (*p* > 0.05). 

The concentrations across all samples were in the range 2.1–4.3 mg of CLA per gram of lipid. The samples exposed to 5 °C rigor temperature and 7 days ageing recorded a CLA concentration mean of 2.71 mg/g initially and a mean of 2.49 mg/g lipid after display time, respectively. The CLA concentrations of the samples exposed to 25 °C rigor temperature and 7 days ageing recorded a mean of 2.92 mg/g initially and 3.13 mg/g of lipid after the display time, respectively. The samples exposed to 5 °C rigor temperature and aged for 14 days had a mean CLA concentration of 2.66 mg/g and after display time at 2.82 mg/g lipid. The reverse was observed with 25 °C rigor temperature samples (2.64-2.80 mg/g lipid), with CLA concentrations not statistically significant. These concentrations were low compared to the findings of Raes et al. [6] and Shantha et al. [30] on beef sirloin and rib eye, who found CLAs ranging from 4.0 to 10 mg/g and 5.8 to 6.8 mg/g lipid, respectively. Many factors can contribute to the reported CLA variations. Adding to the natural biological variations among animals, CLA variation can be attributed to different seasonal, climatic and production systems (e.g., different feeding regimes between countries [1,8]).

The lack of change in CLA concentrations in aged meat experiencing lipid oxidation may be due to the higher stability of CLA compared to other PUFAs containing methylene-interrupted double bonds. This makes the other PUFAs more susceptible to oxidation [30]. The oxidative stability of CLAs compared to the overall lipid oxidative deterioration (TBARS, R_ao,_ R_ad_ proton ratios and fatty acid profiles) discussed in Mungure et al. [22] suggests that these oxidative reactions have limited involvement with CLAs, in agreement with earlier reports [30]. Although other researchers have reported CLAs being oxidized faster and in higher proportion compared with other PUFAs, this is still considerably debatable [31].

A possible mechanistic explanation for CLA stability over ageing and display times is their predominant positional distribution on the glycerol backbone of the triacylglycerol molecule [32]. Investigations on the positional distribution of CLAs on the triacylglycerol molecule have shown that they mainly esterify at the *sn*-2 position [32]. Fatty acids esterified at the *sn*-2 position have been reported to be more stable against oxidation compared to fatty acids esterified at the *sn*-1 and -3 positions [32,33].

The only probable way that oxidative reactions could potentially influence the CLA concentrations (and likely to explain the low numerical fluctuations observed in 25 °C aged for 7 days and 5 °C aged for 14 days samples, Figure 3) is the formation of linoleic acid radicals from PUFAs, which can be converted into CLA by hydrogen donors [3]. The formation of CLA by these chemical reactions would balance out any CLA oxidative loss or breakdown that may have possibly occurred [34].

### 3.2. Effect of Rigor Temperature, Ageing and Display Time on Cholesterol and Its Oxidative Stability in Hot Boned Beef SM Muscle

**Figure 4:**
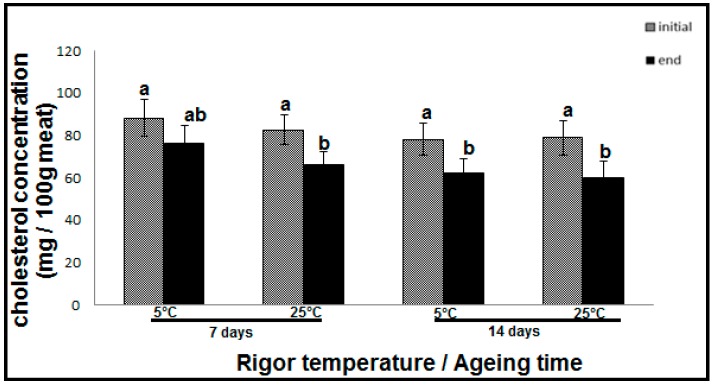
Cholesterol concentrations in hot boned beef SM muscle lipid for samples aged for 7 and 14 days at 2 °C. ^ab^ Values with different letters are significantly different at (*p* < 0.05). Key: “initial” cholesterol concentrations were determined at the start of display time. The “end” cholesterol concentrations were determined at the completion of 7 days of display time.

In this study, the total cholesterol concentrations across all treatments were high, in the range of 60 to 88 mg 100 g^−1^ meat (Figure 4). Figure 4 shows the cholesterol concentrations of 88 mg 100 g^−1^ and 82 mg 100 g^−1^ of meat in samples aged for 7 days and then held at the rigor temperatures of 5 and 25 °C, respectively. In samples aged 14 days, the total cholesterol declined to 76 and 78 mg 100 g^−1^ of meat with rigor temperatures 5 and 25 °C, respectively. Previous studies observed lower total cholesterol concentrations in beef. For example, Chizzolini et al. [13] found concentrations in the range of 47 to 57 mg 100 g ^−1^ meat, and Wheeler et al. [35] reported concentrations of ~63 mg 100g^−1^ meat. The high total cholesterol content may be attributed to natural biological variation among breeds, the selected beef muscle and the farming systems [13]. Cholesterol concentration declined with ageing time (*p* < 0.01). Cholesterol concentration was also affected by display time and its interaction with ageing time (*p* < 0.01). The most significant effect was observed in meat samples after 14 days of ageing. After the display time, up to a 20% decline in cholesterol was observed (Figure 4). Kregel et al. [36] also reported cholesterol decline with ageing time in ground beef. This decrease was also observed in raw chicken sausages aged for 1 week (18–25% decline) and 2 weeks (~14–15% decline) [37]. This decrease in cholesterol concentration may be caused by the generation of numerous oxidation products and possibly related to the structure of the unsaturated alcohol containing the double bond prone to oxidation [19]. The decline in cholesterol concentration was higher after the display time compared to after ageing. This is caused by oxygen and light exposure potentially stimulating the scission of cholesterol to form hydroperoxides (ROOH) [21]. Another cause of the decline in cholesterol may be due to degradative enzymatic reactions [38] forming other oxidative products.

Out of the COPs investigated, 7α and 7β-hydroxycholesterol (7α or β-HC) and 7-ketocholesterol (7-KC) were positively identified (Table 1). Neither 20α-Hydroxycholesterol (20α-HC) nor α- and β-epoxides were detected. This may be due to the fact that 7α-hydroxycholesterol and its epimer 7β-hydroxycholesterol are derived from the immediate decomposition of hydroperoxides, the first products of cholesterol oxidation via autoxidation [17]. The ketone derivate, 7-ketocholesterol, is formed by their subsequent dehydration [14,19]. The relatively short ageing times applied in this study might possibly not have been sufficient enough for the hydroxyperoxy and cholesterol interactions to generate α- and β-epoxides and reactions at carbon 20 for 20α-HC to build up (*p* > 0.05) [9].

Rigor temperature did not have an effect on the generation of COPs (*p* > 0.05). This is shown in Table 1, where samples vacuum packed and aged for 7 days did not have any noticeable COPs, but after display time, both 5 and 25 °C rigor temperature samples showed a detectable, but not statistically significant, increase in the identified COPs (13.5 and 19.4 µg g^−1^ lipid respectively). Ageing and display time had an effect on the formation of oxysterols (*p* < 0.05), increasing their quantity with ageing and display time.

The COPs 7α-β-HC and 7-KC were first identified in samples that had undergone 7 days of ageing and 7 days of display time. This may be due to aerobic conditions triggering the increased production of hydroperoxides from PUFAs. The hydroperoxides are required to initiate more cholesterol oxidation, since this reaction process goes through a similar free radical formation mechanism to that of PUFAs [16].

The samples aged for 14 days pre-display (only exposed to anaerobic conditions) had lower total COP contents (13.0 and 16.2 µg g^−1^ lipid) compared to the post-display samples aged for 7 days (13.5 and 19.4 µg g^−1^ lipid). This again is due to the lack of meat exposure to oxygen during the 14 days ageing in vacuum sealed packaging. This highlights the importance of packaging conditions and the influence of lipid and cholesterol oxidation processes in meat [39].

The samples aged for 14 days under vacuum showed an increase in total COPs up to 75% after 7 days of display time. The ageing process would have reduced the natural antioxidants present in the meat sample, increasing lipid oxidation and formation of oxysterols [15]. Comparing the number of oxysterols formed in this beef study to other animals (for instance with turkey, water buffalo, seafood and pork), one can observe a lower COP content. This can be explained by the low presence of PUFAs in the beef. The PUFA ratios, as discussed in Mungure et al. [22] ranged from 3.7–7.5% across all treatments, whereas turkey meat has been reported to have a total PUFA as high as 26% [39]. The high PUFA content promotes cholesterol oxidation [24], resulting in the high total COPs in turkey meat compared to beef in the present study.

These results were consistent with the findings of Du et al. [39], where vacuum-packing significantly reduced cholesterol oxidation in raw beef. Guardiola et al. [40] also showed the significant importance of vacuum packaging as a means to curb lipid and cholesterol oxidation in spray dried egg.

## 4. Conclusions

The outcome of this study shows that CLA concentrations were not affected by the processing conditions used. The results suggest that higher rigor temperatures may be used to hasten rigor mortis in hot boned SM muscle without compromising CLA levels. Ageing did not affect CLA stability which is a positive outcome; meat can therefore be aged to enhance its tenderness without compromising CLA concentrations. Cholesterol stability was not affected by rigor temperature; however, ageing and aerobic display increased the formation of oxysterols. Ageing and aerobic display treatments did not form high levels of oxysterols and other secondary deleterious secondary COPs, which is a desirable outcome in terms of cholesterol oxidation during the storage of meat and for human health. A limitation of the present study is not examining the progression of the COPs formation upon cooking the meat and this is recommended for future studies.

## Figures and Tables

**Table 1:** COPs identified in hot boned beef SM muscle aged 7 and 14 days (with rigor temperatures 5 °C and 25 °C) (initial) and 7 days of display time (end).

	7 Days Post-Mortem (µg COPs/g Lipid)				14 Days Post-Mortem (µg COPs/g Lipid)			
	Pre-Display (Initial)		Post-Display (End)		Pre-Display (Initial)		Post-Display (End)	
Rigor (°C)	5 °C	25 °C	5 °C	25 °C	5 °C	25 °C	5 °C	25 °C
7α- and 7β-HC	Nd	nd	7.2 ± 0.4 ^ab^	10.6 ± 0.8 ^b^	6.2 ± 0.6 ^b^	8.4 ± 0.7 ^b^	11.8 ± 1.2 ^cd^	12.3 ± 1.6 ^d^
7-KC	Nd	nd	6.3 ± 0.9 ^a^	8.8 ± 0.5 ^ab^	6.8 ± 1.5 ^ab^	7.8 ± 1.8 ^b^	13.5 ± 1.7 ^c^	15.6 ± 0.9 ^c^
α- and β-epoxide	Nd	nd	nd	nd	nd	nd	nd	nd
20α-HC	Nd	nd	nd	nd	nd	nd	nd	nd
Total COPs	-	-	13.5 ± 0.5 ^ab^	19.4 ± 0.6 ^b^	13.0 ± 1.3 ^a^	16.2 ± 1.4 ^b^	25.3 ± 1.6 ^c^	27.9 ± 1.5 ^c^

Mean ± SD, *n* = 6. nd not detected, ^a,b,c,d^ Values with different superscripts within a row are significantly different at *p* < 0.05.
